# Distribution of Microbial Community Structure in Sediment from the Suyeong River and Bay of Busan, Republic of Korea, Determined by 16S rRNA Gene Amplicon Sequencing

**DOI:** 10.1128/MRA.00778-21

**Published:** 2021-11-18

**Authors:** Ilwon Jeong, Junho Lee, Jong-Oh Kim, Seokjin Yoon, Kyunghoi Kim

**Affiliations:** a Department of Ocean Engineering, Pukyong National University, Busan, Republic of Korea; b Research vessel *Nara*, Pukyong National University, Busan, Republic of Korea; c Department of Microbiology, Pukyong National University, Busan, Republic of Korea; d Dokdo Fisheries Research Center, National Institute of Fisheries Science, Pohang, Republic of Korea; Indiana University, Bloomington

## Abstract

Here, we report a 16S rRNA gene amplicon sequence analysis presenting the microbial community in sediments from the Suyeong River and Suyeong Bay, Republic of Korea. The dominant phyla in all sediment samples were *Proteobacteria* (39.69 to 53.62%) and *Bacteroidetes* (29.78 to 33.89%).

## ANNOUNCEMENT

The Suyeong River (SR) is the second-longest river in Busan, Republic of Korea, connecting with an industrial complex and residential and commercial areas from upstream to downstream. In addition, two wastewater treatment facilities (35.188817 N, 129.113647 E and 35.182342 N, 129.120067 E) discharge ca. 14.2 × 10^6^ m^3^/year of treated wastewater into the SR (1). However, during rainfall events, contaminants from the roads and untreated urban sewage wastewater overflow into the SR, flowing to Suyeong Bay (SB) (2). According to the Coastal Pollution Total Volume Control decree, in 2015, SB was designated a Busan Special Management Area to remediate this highly contaminated environment (3). In accordance with the decree, the water quality in the SR and SB has been observed through the River and Marine Environmental Monitoring System (4). Some research has been conducted on the SR and SB, involving water quality, persistent organic pollutants (POPs) and polychlorinated biphenyls (PCBs), and heavy metals (3, 5); however, no research has been conducted related to the microbial community in the sediment from the SR and SB. Therefore, this study provides information about the microbial diversity that characterizes the sediment from the SR and SB.

Sediments from the SR and SB were sampled at five observation points in August 2020 (Table 1). Using a Peterson grab sampler aboard the research vessel *Nara*, surface sediments were sampled from a depth of 15 cm and placed in a sterile ziplock bag. The ziplock bags were kept in a freezer and transferred to Macrogen, Inc. (Seoul, Republic of Korea). According to the manufacturer’s guidelines, 10 g of sediment was used to extract the total DNA using the DNeasy PowerMax soil kit (Qiagen). The Herculase II Fusion DNA polymerase Nextera XT index kit v2 was applied as a 16S rRNA gene amplicon sequencing library kit with the primer set Bakt_341F (5′-CCTACGGGNGGCWGCAG-3′)/805R (5′-GACTACHVGGGTATCTAATCC-3′), following the manufacturer’s instructions. A subsequent limited-cycle amplification step was performed to add multiplexing indices and Illumina sequencing adapters. The PCR for the V3 to V4 region followed a protocol of an initial denaturation at 95°C for 3 min, 25 cycles of 94°C for 30 s, annealing at 50°C for 30 s, and extension at 72°C for 30 s, followed by a final extension at 72°C for 5 min. The 16S rRNA gene amplicon of the sediment sample was sequenced using the Illumina MiSeq platform in 301-bp paired-end format. The adapter sequences were removed and low-quality reads (Q < 20) were filtered using the FASTQ program (6); paired-end data were assembled using FLASH v1.2.11 (7). The operational taxonomic units (OTUs) were clustered using CD-HIT-OTU (over 97% similarity) (8), and the taxonomic assignment was determined using BLAST+ v2.9.0 against the NCBI 16S microbial database. A comparison of the microbial diversity among the samples was conducted using QIIME v1.9.

**TABLE 1:** Description of summary data obtained from the sediment sample in the study

Characteristic	Suyeong River samples			Suyeong Bay samples	
	SY08	SY10	SY13	SY17	SY18
Coordinates	35.179083 N, 129.117983 E	35.170733 N, 129.123067 E	35.163600 N, 129.130283 E	35.156733 N, 129.136483 E	35.153233 N, 129.133967 E
No. of reads	112,819	119,003	115,051	104,991	99,102
No. of OTUs	25,877	28,985	23,733	18,646	18,467
Proportion of bacteria (%)	94.48	93.58	94.45	91.39	90.34
Proportion of archaea (%)	0.00	0.01	0.01	0.03	0.04
SRA accession no.	SRX11551191	SRX11551192	SRX11551193	SRX11551194	SRX11551195

**FIG 1:**
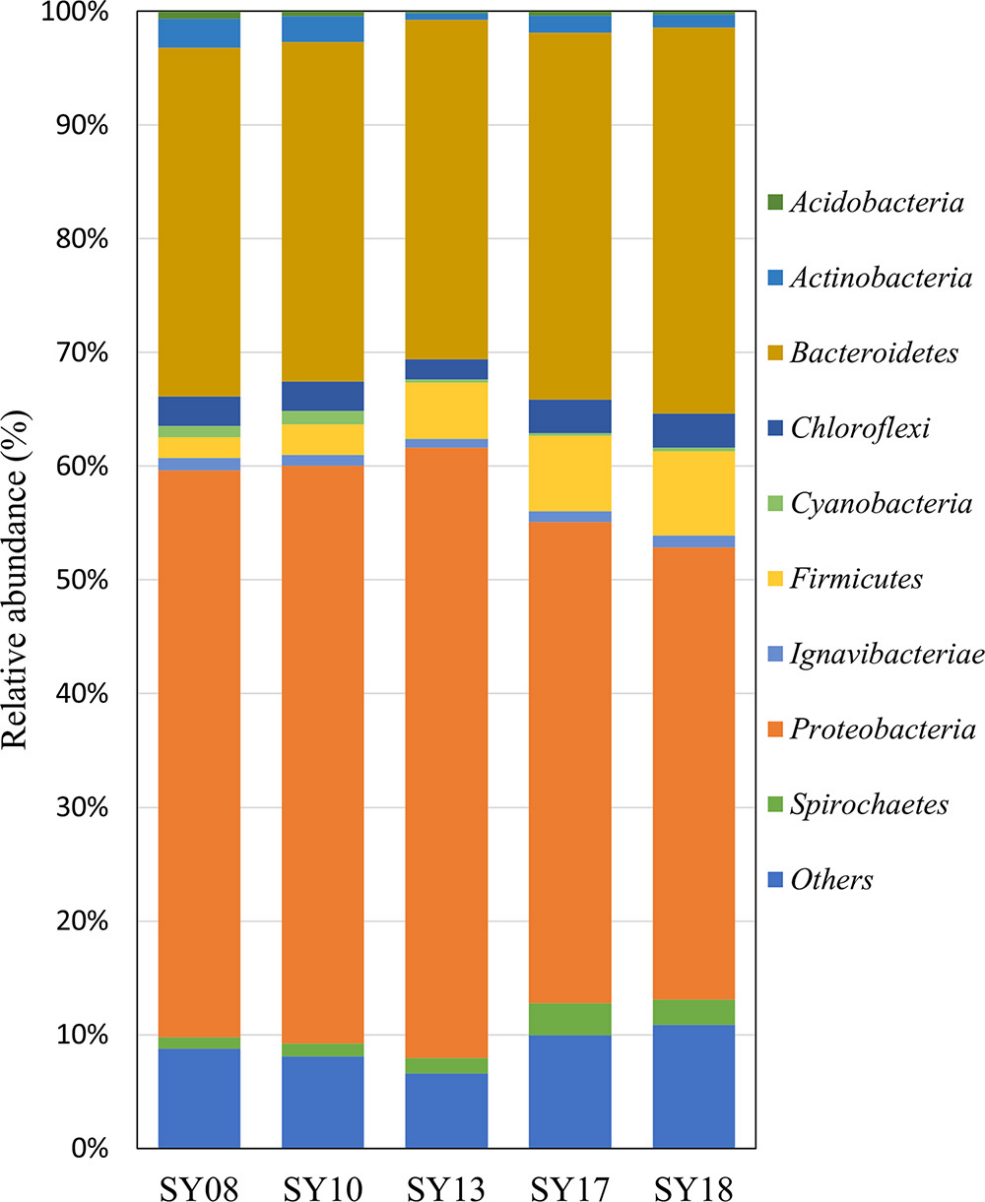
Relative abundance of microbial diversity from the SR and SB based on 16S rRNA gene amplicon sequencing. Phyla with a relative abundance of less than 0.1% and unassigned amplicon sequences were assembled together as “others.”

A total of 550,966 sequence reads and 115,708 OTUs were obtained from 5 samples (Table 1). In total, the OTUs were assigned to 1 archaeal and 22 bacterial phyla, 51 classes, 99 orders, 207 families, and 480 genera. The OTUs at the phylum level (relative abundance, >0.1%) were *Acidobacteria*, *Actinobacteria*, *Bacteroidetes*, *Chloroflexi*, *Cyanobacteria*, *Firmicutes*, *Ignavibacteriae*, *Proteobacteria*, and *Spirochaetes*, as shown in Fig. 1. *Proteobacteria* (relative abundance, 39.69 to 53.62%) was the predominant phylum, followed by *Bacteroidetes* (29.78 to 33.89%), in all sediments. As the sampling location approached SB in the SR, the proportion of *Proteobacteria* decreased from 49.68% to 39.69%, and that of *Bacteroidetes* increased from 29.78% to 33.89%.

### Data availability.

The 16S rRNA gene amplicon sequences from this study are available in the NCBI Sequence Read Archive (SRA) under the accession number PRJNA749664.
